# Protocol for a multi-site, cluster‐randomized, phase III, comparative clinical trial of geriatric assessment of older patients with non‐small‐cell lung cancer: the ENSURE-GA study

**DOI:** 10.1186/s12877-021-02028-w

**Published:** 2021-01-22

**Authors:** Yukari Tsubata, Shun Shinomiya, Koji Inoue, Nobuhisa Ishikawa, Ryota Saito, Kazuhisa Nakashima, Katsuyuki Hotta, Akinobu Hamada, Fumio Nagashima, Yuichi Ando, Satoshi Morita, Kunihiko Kobayashi, Takeshi Isobe

**Affiliations:** 1 Department of Internal Medicine, Division of Medical Oncology & Respiratory Medicine, Shimane University Faculty of Medicine, 89-1 Enya-cho, 693-8501 Izumo, Shimane, Japan; 2 Department of Respiratory Medicine, Saitama Medical University International Medical Center, 397-1 Yamane, 350-1298 Hidaka, Saitama Japan; 3 Department of Respiratory Medicine, Ehime Prefectural Central Hospital, 83 Kasuga-cho, 790-0024 Matsuyama, Ehime, Japan; 4 Department of Respiratory Medicine, Hiroshima Prefectural Hospital, 1-5-54 Ujinakanda, Minami-ku, 734-8530 Hiroshima, Japan; 5 Department of Respiratory Medicine, Tohoku University Hospital, 1-1 Seiryo-cho, Aoba-ku, 980-8574 Sendai, Miyagi Japan; 6 Center for Innovative Clinical Medicine, Okayama University Hospital, 2-5-1 Shikata-cho, Kita- ku, 700-8558 Okayama, Japan; 7 Division of Molecular Pharmacology, National Cancer Center Research Institute, 5-1-1 Tsukiji, Chuo-ku, 104-0045 Tokyo, Japan; 8 Department of Medical Oncology, Kyorin University Hospital, 6-20-2 Shinkawa, Mitaka, 181-8611 Tokyo, Japan; 9 Department of Clinical Oncology and Chemotherapy, Nagoya University Hospital, 65 Tsurumai- cho, Showa-ku, 466-8560 Nagoya, Japan; 10 Department of Biomedical Statistics and Bioinformatics, Kyoto University Graduate School of Medicine, Yoshidahonmachi, Sakyo-ku, 606-8501 Kyoto, Japan

**Keywords:** Geriatric assessment, Lung cancer, Cluster‐randomized trial, Cancer treatment, Patient satisfaction

## Abstract

**Background:**

In Japan, approximately half of all lung cancer patients are aged > 75 years, and the proportion of older patients is increasing. In older patients, it is necessary to consider comorbidities and concomitant drug use to ensure optimal cancer treatment; however, geriatric assessment (GA) is not widely performed. We plan to conduct a study (ENSURE-GA) of GA in older lung cancer patients to determine whether GA with intervention improves patient satisfaction with their treatment.

**Methods:**

The study will be a phase III comparative clinical trial with a cluster-randomized design, and it will be conducted at 81 sites distributed throughout Japan. Approximately 1000 lung cancer patients aged ≥ 75 years will be enrolled in the study. All participants will undergo a standardized GA before starting treatment (using an iPad). At the intervention sites, the GA results and intervention method recommended on the basis of the GA results will be returned as an instant report to guide the physician’s choice of intervention. At the control sites, the physician will decide on interventions based on standard practice. All participants will complete a patient satisfaction survey before treatment initiation (after the GA) and 3 months later.

**Discussion:**

The purpose of the ENSURE-GA study is to evaluate whether GA with interventions improves patient satisfaction with treatment outcomes. The study may lead to the increased use of GA and improved treatment of cancer in older adults. The results will also be used to prepare guidelines for treating older cancer patients and will provide a foundation for the development of a standardized geriatric oncology system.

**Trial registration:**

The study has been registered in the University Hospital Medical Information Network database (no. UMIN000037590). The registration date is August 4, 2019, and the protocol version is 2.0. (https://upload.umin.ac.jp/cgi-open-bin/ctr/ctr_view.cgi?recptno=R000042853.)

## Background

Aging is a global phenomenon among patients with cancer. According to the National Cancer Center Information Service, people aged > 65 years currently account for 73 % of the total patients with cancer, and this proportion continues to increase, especially in Japan, which is particularly important. In 2015, among males, 79 % of patients with lung cancer were aged > 65 years and 48 % were aged > 75 years, while among females, 78 % of patients with lung cancer were aged > 65 years and 50 % were aged > 75 years. Older cancer patients are diverse; thus, in conjunction with their primary treatment, supportive and palliative treatments should be provided based on the results of a geriatric assessment (GA). The GA should evaluate aging-related deterioration in physiological function, comorbidities, and polypharmacy to ensure safe and effective chemotherapy.

In 2018, the American Society of Clinical Oncology published the article “Practical Assessment and Management of Vulnerabilities in Older Patients Receiving Chemotherapy: ASCO Guideline for Geriatric Oncology” [1], which called for the development of detailed, evidence- and/or consensus-based GA tools, and interventions for rehabilitation, improvement of nutritional state, and adjustment of concomitant drugs. In Japan, GA is not currently performed in clinical practice because there is no standard method for performing GA, it takes considerable time, and there is little evidence of its usefulness in making decisions regarding cancer treatment [2]. Older cancer patients are currently not administered individualized treatment, and they may be either under- or over-treated [3]. To improve the current situation, there is a need to develop a simple system for performing GA, establish a GA environment, and determine the usefulness of GA in clinical practice. Therefore, we plan to conduct a study (ENSURE-GA) of GA in older patients with lung cancer to evaluate whether performing GA along with intervention improves patient satisfaction with their treatment. We believe that the study findings will improve the penetration rate of GA assessment in Japan and promote the provision of personalized medicine to older patients with cancer.

## Methods

### Study objective, design, and setting

The primary objective of the ENSURE-GA study is to evaluate the effect of an intervention based on a standardized GA on patient satisfaction with their treatment. This study will be a phase III comparative clinical trial with a cluster-randomized design and will be conducted in 81 medical facilities that have been selected based on the following four factors: (i) number of beds ( ≧ 200 beds or < 200 beds); (ii) whether or not designated as a cancer hospital by the Japanese Ministry of Health, Labor, and Welfare; (iii) region (divided into 6 regions, and these regions were evenly allocated with participating facilities from each region); and (iv) whether GA is usually included in medical treatment. Each facility will be randomly allocated in order of enrollment to the intervention or non-intervention group in a 1:1 ratio.

The primary endpoint will be the change in patient satisfaction according to the Health Care Communication Questionnaire (HCCQ) questionnaire between the GA and 3 months later. The secondary endpoints will be (i) the change in the quality of life; (ii) the treatment completion rate; (iii) the proportion of participants showing improvement by at least one grade in their Eastern Cooperative Oncology Group performance status (ECOG-PS); (iv) the relationship between the pre-chemotherapy risk assessment results and adverse events, evaluated separately for cytotoxic anticancer agents, molecular-targeted agents, and immune checkpoint inhibitors; and (v) the proportion of screened patients who had vulnerability factors in at least one of the GA tools and are enrolled in the study.

**Fig. 1:**
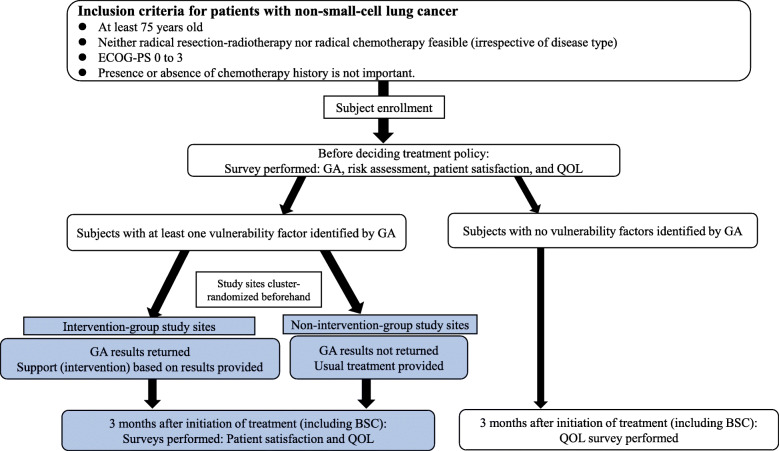
Study flowchart summarizing the study procedures. Abbreviations: BSC, best supportive care; ECOG-PS, Eastern Cooperative Oncology Group performance status; GA, geriatric assessment; QOL, quality of life

We have built a GA tool as an iPad application and have developed a system that displays GA results and recommendations for related interventions. Using this system, GA is performed for each participant. In the intervention facilities, participants’ GA results and the intervention method recommended on the basis of these results will be displayed immediately on the iPad in the form of a report. Using this report, the responsible physician will explain the GA results and the intervention method to the participant (Table 1). In the non-intervention facilities, the physician will perform a GA, but the results and the intervention method recommended based on these results will not be displayed on the iPad. The physician will decide on treatment solely on the basis of the ECOG-PS and multiple-organ function, as is currently the standard for lung cancer treatment in Japan. Patients who are analyzed for the primary endpoint are those with one or more vulnerability factors identified using the GA tools. All participants will complete a patient satisfaction survey before treatment initiation and 3 months later (Fig. 1). The ENSURE-GA study is an intergroup trial, and the North East Japan Study Group and CS-Lung are working together to conduct the trial.

**Table 1:** Geriatric assessment tools and examples of possible interventions based on the assessment results

Evaluated items	Recommended GA tools	Example of intervention (support) method based on the GA results
Physical function: Falls	• Lawton IADL • “How many times have you fallen during the last 6 months?”	• Referral to an occupational therapist and/or physiotherapist • Prevention of falls • Evaluation of safety of home and garden
Complications, polypharmacy	• CCI • Number of drugs in regular use	• Encouraging involvement of family members in management of complications • Collaboration between the primary-care physician and a geriatrician • Consulting a pharmacist regarding reducing the number of medications
Cognitive function	• Mini-Cog	• Evaluation of decision-making capability • Selection of someone with power of attorney, if necessary • Collaboration between a geriatrician and a cognitive function specialist • Prevention of delusions, by adjusting medication
Depressive state	• GDS	• Referral to a psychiatrist • Promoting participation in social activities • Prescription of an antidepressant
Nutrition	• BMI below 21 kg/m^2^	• Referral to a dietician • Provision of support for preparing meals
Social support	• Do you have key persons, family, or friends regarding cancer treatment?	• Referral to a social worker

*BMI* body mass index; *CCI* Charlson Comorbidity Index; *GA* geriatric assessment; *GDS* geriatric depression scale; *IADL* Instrumental Activities of Daily Living Scale; *Mini-Cog* Mini-Cog screening for cognitive impairment in older adults

Ethics approval will be obtained from the ethics committee of each participating institution. All patients will sign an informed consent form before inclusion. The study has been registered in the University Hospital Medical Information Network database (no. UMIN000037590).

### Inclusion and exclusion criteria

Participants will be required to meet all of the following inclusion criteria:

- histologically and/or cytologically confirmed to have non-small-cell lung cancer,
- radical resection-radiotherapy and radical chemotherapy are not feasible,
- age at least 75 years,
- ECOG-PS of 3 or less, and.
- provide informed consent for participation.

Patients with any of the following factors will be excluded from the study:

- dementia and aggressive cancer treatment,
- psychiatric disorders that could interfere with participation, and.
- study participation is judged by the responsible physician to be inappropriate for any other reason.

### GA tools, questionnaires, and chemotherapy risk assessments

A list of GA tools and recommended intervention methods is shown in Table 1.

GA tools to be used include:

- Lawton Instrumental Activities of Daily Living Scale,
- history of falls within the past 6 months,
- Charlson Comorbidity Index (CCI),
- Mini-Cog screening for cognitive impairment in older adults, and.
- Geriatric Depression Scale (GDS).

The number of medications used, body mass index, and social support will also be assessed as part of the GA.

Questionnaires to be employed include:

- Patient Satisfaction Survey in HCCQ, and.
- European Organization for Research and Treatment of Cancers QLQ-C15-PAL.

Chemotherapy risk assessments to be performed are as follows:

- Cancer and Aging Research Group score [4, 5], and.
- Geriatric 8 health status screening tool.
- Collect grade 3 or higher adverse events with Common Terminology Criteria for Adverse Events v5.0.

### Outcome

The primary outcome will be the change in the level of patient satisfaction according to the HCCQ questionnaire between the pre-treatment assessment and the post-treatment assessment conducted 3 months later.

## Sample size

For calculating the required number of subjects, first, the number needed for randomization of individual subjects will be calculated, followed by the number needed for cluster randomization. With reference to a report by Mohile et al. [6], it is predicted that the mean scores in the HCCQ Patient Satisfaction Survey before chemotherapy will be 23.3 and 22.2 in the intervention and non-intervention groups, respectively. Considering the common standard deviation for this variable to be 4.5, the two-sided α error to be 5 %, and the detection power to be 80 %, the required number of subjects is calculated to be 526.

Taking the required number of subjects for separate randomization to be *n*, the required number for cluster randomization to be *n’*, the cluster size to be *m*, and the intra-cluster correlation coefficient to be *ρ*, the formula is as follows:

$$n' = ([m - 1] \rho + 1) n$$

Using the above formula, taking *n* to be 526, *m* to be 15, and *ρ* to be 0.05 yields a value of 895 for *n’*, and a required number of clusters of 60; therefore, the number of study sites has been set at a minimum of 60. We assumed that 10 % of prospective participants would have at least one exclusion criterion, and thus the minimum number of participants required is 985.

In addition, in a previous study by our research group, it was found that, among lung cancer patients aged over 75 years, 97 % showed at least one vulnerability factor (unpublished data). Taking this into consideration, the sample size has been set at 1020. Eighty-one medical facilities (study sites) distributed throughout Japan have been selected for the study. We aim to enroll 15 participants per site.

### Statistical analysis

A mixed-effects model will be used to perform intergroup comparisons taking intra-cluster correlation into consideration, and the 95 % confidence interval will be calculated. Statistical analyses are performed using the Statistical Analysis System, version 9.4 (SAS Institute Inc., NC, USA).

## Discussion

GA for elderly cancer patients requires a multifaceted assessment, is complicated, and takes considerable time, and hence it has largely been avoided. For this study, detailed GA tools and intervention methods have been identified, and a simplified and standardized iPad system will be used to record the data. If the iPad GA system is shown to be feasible, it may encourage physicians to use this method of GA in general clinical practice and to base their interventions on the GA results. In 2019, Mohile et al. [6] reported that GA is effective for improving communication between patients and healthcare workers; however, only five phase III, comparative, clinical trials, including the ENSURE-GA study, have been conducted to determine the usefulness of GA, and there have been no trials that have evaluated interventions based on the results of a GA [1]. Japan has one of the highest levels of societal aging in the world, and GA is not currently used in standard practice to guide the treatment of older cancer patients.

In conclusion, the ENSURE-GA study will determine the usefulness of GA for individualizing the treatment of older patients with lung cancer. If the intervention is shown to be effective, it may lead to the increased use of GA and improved treatment of older cancer patients. The study has the potential to have a major impact not only on the management of lung cancer but also on the management of a wide range of cancer types. The results will also be used to prepare guidelines on treating older cancer patients and will establish a foundation for the development of a standardized geriatric oncology system.

## Data Availability

All data is supported by and stored on the portal site of the Practical Research for Innovative Cancer Control Management Office created by the Japan Cancer Research Project, one of the projects promoted by the Japan Agency for Medical Research and Development, https://portal.jcrp-primo.jp/.

## Abbreviations

BMI: Body mass index
BSC: Best supportive care
CCI: Charlson Comorbidity Index
ECOG-PS: Eastern Cooperative Oncology Group performance status
GA: Geriatric assessment
GDS: Geriatric Depression Scale
HCCQ: Health Care Communication Questionnaire
IADL: Instrumental Activities of Daily Living Scale
Mini-Cog: Mini-Cog screening for cognitive impairment in older adults
QOL: Quality of life
